# Enzyme-assisted valorization of agro-industrial byproducts for sustainable and efficient broiler production

**DOI:** 10.14202/vetworld.2026.782-804

**Published:** 2026-02-26

**Authors:** S. Sugiharto, F.R. Handayani, D.N. Adli, M.M. Sholikin, T. Ujilestari

**Affiliations:** 1 Department of Animal Science, Faculty of Animal and Agricultural Sciences, Universitas Diponegoro, Semarang, Indonesia; 2 Smart Livestock Industry Study Programme, Department of Feed and Animal Nutrition, Faculty of Animal Science and Technology, Universitas Brawijaya, Malang, 65145, Indonesia; 3 Research Center for Animal Husbandry, National Research and Innovation Agency, Bogor, Indonesia

**Keywords:** agro-industrial byproducts, broiler chicken feed, broiler performance, enzyme supplementation, feed efficiency, non-starch polysaccharides, palm kernel cake, sustainable poultry production

## Abstract

Agro-industrial byproducts, including distillers dried grains with solubles, sunflower seed meal, palm kernel cake, sweet orange peel meal, brewers’ dried grain, and various fruit and vegetable processing wastes, represent abundant, low-cost alternatives to conventional feed ingredients such as corn and soybean meal in broiler chicken diets. Their incorporation supports sustainability by reducing feed costs, alleviating food-feed competition, and promoting circular bioeconomy principles through waste valorization. However, the presence of complex non-starch polysaccharides, lignocellulosic structures, phytate, mannans, and other antinutritional factors often limits nutrient digestibility, impairs intestinal health, and compromises broiler growth performance when these byproducts are included at higher levels. Exogenous enzyme supplementation, particularly phytase, protease, xylanase, β-glucanase, β-mannanase, cellulase, and multi-enzyme complexes, has emerged as an effective strategy to overcome these limitations. Enzymes hydrolyze indigestible components, reduce digesta viscosity, improve nutrient availability (dry matter, crude protein, energy, and phosphorus), enhance intestinal morphology (increased villus height to crypt depth ratio), modulate gut microbiota toward beneficial populations such as Lactobacillus and Bifidobacterium, and mitigate inflammatory responses. These improvements enable substantially higher inclusion levels of byproducts, up to 50% substitution of conventional ingredients in some cases, without negative effects on body weight gain, feed conversion ratio, or overall performance. Reported performance gains include 1%–16% increases in weight gain, 2%–11% in feed intake, and 1%–26% reductions in feed conversion ratio, depending on byproduct type, enzyme combination, and inclusion level. Economically, enzyme supplementation often offsets its initial cost through better feed efficiency, resulting in lower production cost per kilogram of broiler meat (reductions of 7%–12% in several studies). Environmentally, the approach decreases reliance on high-carbon-footprint crops, reduces manure emissions, lowers greenhouse gas contributions from feed production, and supports waste minimization. Challenges remain, including variability in byproduct composition, enzyme specificity and stability, seasonal quality fluctuations, and occasional inconsistent results across trials. This review concludes that strategic enzyme supplementation offers a practical, science-based pathway to increase the sustainable and efficient utilization of agro-industrial byproducts in broiler production. Future efforts should focus on tailored multi-enzyme formulations, integration of omics technologies for precise matching of enzymes to specific byproducts, and large-scale commercial validation to facilitate wider industry adoption.

## INTRODUCTION

Currently, the sustainability of broiler production is complicated by fluctuations in the availability and price of feed caused by climate change and geopolitical issues. Climate change intensifies the vulnerability of the broiler feed supply chain. Extreme weather adversely impacts crop production, affecting the availability of feed ingredients such as corn and soybean. This results in increased feed costs and ultimately undermines the economic viability of broiler production [1]. Wang *et al*. [2] showed that between 1998 and 2017, climate change-induced droughts in northeast China led to a 15.6 % reduction in corn production and a 20.4 % reduction in soybean production. Moreover, geopolitical tensions, such as international conflicts and economic sanctions, exacerbate vulnerabilities within the broiler feed supply chain, causing increased costs and delivery delays [3].

The use of agro-industrial byproducts as alternative feed ingredients for broilers has gained considerable interest due to their potential to enhance poultry production sustainability. Agro-industrial byproducts are materials remaining after the processing of crops and foods, and they have the potential to reduce feed costs and minimize the competition between food and feed resources [4, 5]. Agro-industrial byproducts, such as fruit juice industry leftovers, oilseed industrial byproducts, distillers’ grain byproducts, and byproducts from the vinification and olive oil industries, can be viable substitutes for conventional feedstuffs, such as corn and soybean meal [6, 7]. These byproducts are often rich in bioactive compounds, which can promote the health and well-being of broilers, offering both nutritional benefits and functional feed capabilities. Nevertheless, agro-industrial byproducts may cause nutritional variability and anti-nutritional factors in broiler chickens [6].

Enzyme supplementation is increasingly being recognized as a valuable strategy for optimizing broiler diets, especially when incorporating agro-industrial byproducts. The use of exogenous enzymes in broiler diets has become a common practice due to their ability to enhance nutrient digestibility and growth performance. Kwak *et al*. [8] demonstrated that enzyme supplementation can effectively allow distillers dried grains with solubles to serve as a cost-effective fiber source without compromising growth performance. Enzyme supplementation also allowed for an increase in sweet orange peel meal (OPM) up to 25 % in broiler diets without adversely affecting final body weight, weight gain, feed intake, and protein digestibility compared to 20 % sweet OPM without enzyme [9]. One study on the use of distillers dried grains with solubles in broiler diets highlighted that supplementation with enzymes improved the digestibility of dry matter, crude protein, and gross energy [8, 10]. Enzymes can enhance nutrient digestibility; however, the selection of specific enzymes and their concentrations is critical for optimizing the use of agro-industrial byproducts in broiler chicken diets.

**Figure 1:**
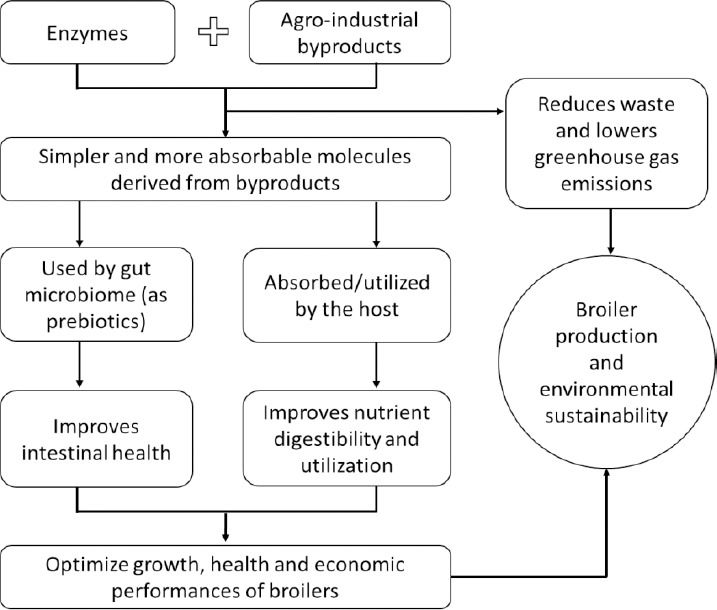
Schematic illustration of how enzyme supplementation promotes sustainability in broiler chicken production while delivering environmental benefits. The central role of enzymes enables higher utilization of agro-industrial byproducts, improves feed efficiency, reduces reliance on conventional high-impact feed ingredients, lowers greenhouse gas emissions and resource use, enhances animal health and performance, and supports circular bioeconomy principles through waste valorization [Source: The figure was prepared by the authors].

Overall, the use of enzyme-supplemented agro-industrial byproducts is beneficial for optimizing growth performance, health, and economic efficiency while reducing waste and greenhouse gas emissions. Therefore, this practice promotes the sustainability of broiler production and the environment (Figure 1).

Although several studies on enzyme supplementation exist, no integrated review has critically assessed its sustainability, economic feasibility, and One Health implications on broiler chickens fed agro-industrial byproducts.

Therefore, this review elaborates the present state of knowledge about the use of enzymes to improve the nutritional value and use of agro-industrial byproducts in broiler diets. The mechanisms by which enzyme supplementation increases nutritional digestibility, intestinal health, and growth performance in broilers fed with diets containing these byproducts are discussed.

## REVIEW METHODOLOGY

This narrative review was conducted to synthesize and critically evaluate the existing literature on the enzyme-assisted utilization of agro-industrial byproducts in broiler chicken diets, with a focus on nutritional enhancement, performance outcomes, gut health, economic implications, and sustainability aspects.

Literature was identified through comprehensive searches in major scientific databases, including PubMed, Scopus, Web of Science, Google Scholar, and relevant animal science-specific repositories (e.g., CAB Abstracts). The search was performed using combinations of the following keywords and phrases: “broiler” OR “broiler chicken” OR “poultry”, “enzyme” OR “exogenous enzyme” OR “phytase” OR “xylanase” OR “β-mannanase” OR “protease” OR “multi-enzyme”, “agro-industrial byproducts” OR “agricultural byproducts” OR “distillers dried grains with solubles” OR “DDGS” OR “palm kernel cake” OR “sunflower meal” OR “orange peel” OR “fruit waste” OR “brewers dried grain”, “feed” OR “diet” OR “nutrition” OR “performance” OR “digestibility” OR “sustainability”. No strict date restrictions were applied to capture foundational and recent developments, though emphasis was placed on publications from the last 20 years (approximately 2005–2025) to reflect current enzyme technologies and byproduct applications. Additional relevant studies were identified through manual screening of reference lists from key review articles, meta-analyses, and high-impact papers in poultry nutrition.

Inclusion criteria focused on peer-reviewed original research articles, reviews, and conference proceedings published in English that reported in vivo effects of exogenous enzyme supplementation in broiler diets containing agro-industrial byproducts. Studies were prioritized if they evaluated performance parameters (e.g., body weight gain, feed intake, feed conversion ratio), nutrient digestibility (e.g., dry matter, crude protein, energy, phosphorus), intestinal health (e.g., morphology, microbiota), carcass traits, economic outcomes, or environmental impacts. In vitro studies on enzyme activity or byproduct processing were included only when they directly supported in vivo findings or mechanisms. Exclusion criteria eliminated non-poultry studies, non-broiler species, studies without enzyme supplementation, purely theoretical papers, and non-peer-reviewed sources (e.g., industry reports, theses without published validation).

A total of over 100 relevant publications were screened, with approximately 80 key studies selected for detailed synthesis based on relevance, methodological quality, and contribution to the review objectives. Selection was not strictly systematic (as per PRISMA guidelines for systematic reviews) but followed a narrative approach to allow flexible integration of diverse evidence types, including experimental trials, mechanistic studies, and applied research. No formal meta-analysis was performed due to heterogeneity in byproduct types, enzyme formulations, inclusion levels, experimental designs, and outcome measures.

Data extraction focused on key elements: byproduct type and inclusion level, enzyme(s) used and dosage, experimental design (e.g., bird age, duration, replicates), main findings on performance, digestibility, health, and sustainability metrics, as well as reported limitations or challenges. Findings were organized thematically (e.g., by enzyme class, byproduct category, or outcome type) to facilitate critical discussion of mechanisms, benefits, constraints, and research gaps. All cited studies were evaluated for scientific rigor, including appropriate controls, statistical analysis, and reporting transparency, though no formal quality scoring tool was applied given the narrative nature of the review.

## FEED AS A MAJOR FACTOR IN SUSTAINABLE BROILER PRODUCTION

Feed cost is a significant factor that affects the profitability and sustainability of the broiler industry. Feed costs account for the largest proportion of overall production costs, often reaching up to 70 % of total expenditure [11]. Hence, optimizing feed costs is critical for achieving economic sustainability in broiler production. In addition to feed costs, the availability and quality of feed greatly influence the sustainability of the broiler industry [12].

Currently, the availability of conventional feed ingredients, such as soybean meal and yellow corn, often fluctuates due to increasing global demand for these products, especially in developing countries. These conventional feed ingredients are also in high demand for other livestock species and human consumption, leading to increased competition and potential price volatility. Feed price fluctuations are closely related to factors such as climate change, geopolitical conflicts, and international trade dynamics [1, 3].

Studies have shown that climate change is causing more frequent and severe extreme weather events, leading to substantial fluctuations in crop yields (e.g., corn and soybean). Geopolitical conflicts can intensify this situation by disrupting supply chains and trade flows, resulting in sudden price fluctuations in global feed markets. Disruptions in global supply chains due to geopolitical events can lead to increased costs, delivery delays, and even production shutdowns [3]. This can result in shortages of essential feed ingredients and price volatility for the poultry industry. The shift in agricultural production priorities toward biofuel crops, driven by high oil prices and supportive agricultural policies, further complicates the situation [13].

Feed efficiency, which refers to how effectively broilers convert feed into body mass, plays a critical role in broiler industry feed cost management. In addition to improving rearing conditions, feed efficiency can be enhanced through dietary modifications, such as the addition of feed additives that improve nutrient absorption and utilization [12]. Broiler strains have been developed through genetic selection to achieve rapid growth and lower feed consumption per unit of meat produced. This approach represents a significant strategy for improving broiler chicken feed efficiency [14].

The broiler industry has shifted toward antibiotic-free production due to increasing concerns about antibiotic resistance. This shift necessitates the exploration of antibiotic alternatives that can improve growth performance, feed efficiency, and overall health [15]. Considering the increasingly expensive and volatile feed costs, the use of agro-industrial byproducts as alternative sources of protein and energy is becoming increasingly crucial for the sustainability of the broiler chicken industry.

## DEFINITION AND TYPES OF AGRO-INDUSTRIAL BYPRODUCTS

Agro-industrial byproducts are secondary materials generated during agricultural and food processing activities, including fruit processing, crop cultivation, and other food manufacturing processes [16, 17]. These materials are frequently regarded as waste and, if not properly managed, can create environmental challenges. Agro-industrial byproducts are primarily composed of lignocellulosic materials, such as cellulose, hemicellulose, and lignin [18]. They are also rich in bioactive compounds, including phenolic compounds, organic acids, and carotenoids [19, 20], and contain valuable nutrients such as complex carbohydrates, proteins, lipids, vitamins, minerals, and dietary fibers [21].

Historically viewed as industrial waste, these byproducts are now increasingly recognized as valuable resources for various applications [22]. This change in perspective has stimulated interest in reincorporating them into production chains, either as feed ingredients or as sources of health-promoting bioactive compounds [16]. Their utilization supports the principles of a circular bioeconomy, delivering benefits to broiler producers and consumers while reducing waste and environmental impact [16, 19].

Agro-industrial byproducts encompass a wide range of materials from agricultural and food processing. Key categories include:

1. Fruit and vegetable processing wastes, such as grape pomace, which are rich in phenolic compounds and exhibit high antioxidant activity [23]. Residues from fruit processing industries can be reintroduced into the food chain as sources of bioactive compounds [16].
2. Lignocellulosic materials, common in many agro-industrial wastes, including wheat bran, soybean bran, and spent coffee grounds (SCA) [24]. These are typically high in cellulose, hemicellulose, and lignin [18, 25].
3. Wastes from winemaking and olive oil production, which contain substantial amounts of polyphenolic compounds [26] and generate large volumes of high-pollutant wastewater [27].
4. Calcium-rich wastes from biological sources (e.g., finger citron, shells of cockle, mussel, oyster, and eggshell) and inorganic sources (e.g., gypsum, dolomite, and sludge) [28].
5. Animal and fisheries refuse and byproducts [29].

Baldi and Merener [30] reported that approximately 57 % of global corn production and 70 % of global soybean production (in the form of meal) are used as livestock feed, particularly for poultry and pigs. Global corn production reaches 1,190 million metric tons, with nearly 684 million metric tons directed to livestock feed. Soybean production totals 363 million metric tons, of which 255 million metric tons serve as a protein source for livestock. With the growing global livestock population, demand for corn and soybean meal as energy and protein sources is projected to rise further, reaching an estimated 795 million metric tons for corn and 290 million metric tons for soybean meal by 2032 [30]. Diversifying alternative energy and protein sources is therefore essential to meet future livestock needs, particularly for broiler chickens. Specific global data on corn and soybean meal use in broiler production remain limited.

Worldwide, agricultural activities generate approximately 140 billion metric tons of biomass waste annually, offering substantial potential as an alternative feed resource [31]. Southeast Asia, with its major commodity crops (rice, palm oil, sugar cane, and corn), holds significant promise for byproduct valorization. Indonesia, the largest country in the region, produces an estimated 10.7 million tons of agro-industrial byproducts annually, including rice husks, cocoa pods, coconut shells, oil palm shells, and corncobs [32]. As a leading palm oil producer, Indonesia generates over 4.16 million tons of palm kernel cake each year [33], representing a major opportunity as an alternative energy source for farm animals.

Agro-industrial byproducts have both positive and negative environmental implications. Large-scale waste generation from food industries, especially fruit processing, imposes a considerable environmental burden [16]. Fruit processing waste contributes notably to greenhouse gas emissions (8%–10% of total annual global emissions) and has a significant water footprint [34]. At the same time, there is growing interest in reintroducing these byproducts into value chains as broiler feed ingredients or sources of bioactive compounds, thereby supporting a circular bioeconomy and waste reduction [16].

Interestingly, while agro-industrial byproducts can contribute to pollution and waste accumulation, they also present opportunities for sustainable innovation. For example, biosurfactants derived from agro-industrial waste are valued for their sustainability and environmental compatibility [35] and can be applied in bioremediation, food processing, and agriculture, aligning with waste minimization goals [35]. Similarly, lignocellulosic residues from soyhulls have been used to produce biodegradable films as an eco-friendly alternative to conventional plastic packaging, advancing circular economy principles [36].

In the broiler industry, incorporating agro-industrial byproducts into chicken diets represents a sustainable approach that simultaneously addresses environmental concerns and nutritional requirements. Given their nutritional composition, these byproducts serve as viable alternatives to conventional feed ingredients. This practice reduces food-feed competition, enhances environmental sustainability, and provides economic and social benefits by adding value to materials previously considered waste [14, 27, 28]. The environmental impact of agro-industrial byproducts is multifaceted: while they can contribute to pollution and waste issues, they also offer pathways for sustainable practices and innovative products.

## USE OF AGRO-INDUSTRIAL BYPRODUCTS IN BROILER CHICKEN RATIONS

Agro-industrial byproducts have emerged as promising alternatives in broiler chicken diets, providing potential benefits for health, performance, sustainability, and cost-effectiveness. Numerous studies have demonstrated the successful inclusion of various byproducts in broiler rations. For example, supplementing broiler diets with up to 10 % extruded flaxseed meal can increase the omega-3 fatty acid content in meat, although higher inclusion levels may impair growth performance and oxidative stability [37]. Similarly, replacing two-thirds of the diet with milk thistle seeds in starter and grower phases improved body weight, feed conversion ratio (FCR), and the polyunsaturated fatty acid profile in breast and leg muscles [38]. These findings indicate that agro-industrial byproducts hold considerable promise for enhancing poultry production performance, meat quality, and overall sustainability. However, optimal inclusion levels and appropriate processing methods must be carefully determined to maximize advantages while minimizing any adverse effects [6].

Despite these opportunities, several constraints limit the widespread use of agro-industrial byproducts in broiler rations. The bioavailability and digestibility of nutrients are often low due to the presence of complex polymers and anti-nutritional factors [6]. Bioconversion techniques, such as solid-state fermentation (SSF), can degrade these compounds and improve nutrient availability [6], but scaling up such processes introduces additional complexity and cost, which may hinder practical adoption.

Another major limitation is the inconsistent composition and quality of agro-industrial byproducts. Variability in nutrient content, moisture, and anti-nutritional factors makes it difficult for producers to formulate nutritionally balanced rations that consistently meet the requirements of broilers [6, 7]. In some cases, certain components—such as raffinose-series oligosaccharides present in various plant-based byproducts—can exert negative effects on animal health and productivity [39]. This underscores the importance of careful selection, preprocessing, and quality control to ensure beneficial rather than detrimental outcomes.

Furthermore, technical and economic barriers exist, particularly when byproducts are intended as alternative protein sources to replace conventional ingredients like soybean meal [40]. The costs associated with collection, transportation, drying, processing, or detoxification of some byproducts can offset their apparent economic advantages, limiting their competitiveness in commercial feed formulations.

Taken together, agro-industrial byproducts offer significant potential to support sustainable broiler production by reducing reliance on conventional feedstuffs, lowering costs, and promoting circular economy principles. Nevertheless, their practical implementation is constrained by challenges related to nutrient bioavailability, compositional variability, processing requirements, and economic feasibility. Continued research is therefore essential to optimize bioconversion and pretreatment methods, standardize byproduct quality parameters, establish reliable inclusion guidelines, and comprehensively evaluate long-term impacts on broiler welfare, performance, product quality, and production economics.

## ENZYMES AND THEIR APPLICATION IN BROILER CHICKEN PRODUCTION

Enzymes are specialized proteins that act as biological catalysts, accelerating chemical reactions in living organisms without being consumed in the process [41]. They are systematically classified by the Enzyme Commission (EC) number system into seven main classes based on the type of reaction catalyzed: (1) Oxidoreductases, which facilitate oxidation-reduction reactions involving electron transfer and play key roles in metabolic pathways and energy production. (2) Transferases, which transfer functional groups (e.g., methyl or phosphate groups) between molecules and are essential in metabolism and signal transduction. (3) Hydrolases, which catalyze the hydrolysis of chemical bonds and are critical for digestion, breaking large molecules (e.g., proteins) into smaller units such as amino acids. (4) Lyases, which add or remove groups to form or break double bonds without hydrolysis or oxidation, contributing to metabolic and biosynthetic pathways. (5) Isomerases, which rearrange atoms within a molecule to produce isomers and maintain biochemical diversity. (6) Ligases, which join two molecules with the hydrolysis of a high-energy bond (typically in ATP), supporting processes such as DNA replication and repair. (7) Translocases, which move ions or molecules across membranes or separate them within cellular compartments, maintaining ion gradients and organelle function [42].

This classification framework helps researchers understand enzyme mechanisms and tailor their applications in nutrition, biotechnology, and animal production.

In broiler chicken production, exogenous enzymes are widely used to enhance the nutritional value of feed, counteract anti-nutritional factors, and improve overall performance and health. The most commonly applied enzyme classes in poultry diets include: (1) Phytase, the most extensively used enzyme, which hydrolyzes phytic acid (phytate) in plant-based ingredients, thereby improving phosphorus availability, feed utilization, and reducing environmental phosphorus excretion [43]. (2) Protease, which degrades protein anti-nutrients and enhances protein digestibility, leading to better nutrient absorption and broiler performance [44]. (3) Non-starch polysaccharide (NSP)-hydrolyzing enzymes, such as xylanase and β-glucanase, which break down viscous fiber components, reduce intestinal digesta viscosity, improve nutrient digestion, and enhance growth and feed efficiency [45, 46]. (4) β-Mannanase, which specifically targets mannans in feed ingredients, improving nutrient digestibility, supporting beneficial gut microbiota, and boosting immune responses in broilers [47]. (5) Fibrolytic enzymes (e.g., endo-xylanase, endo-glucanase, cellulase, and hemicellulase), which degrade cellulose and hemicellulose into simpler sugars, facilitating the utilization of fibrous feed materials and improving nutrient absorption and animal performance [46].

Collectively, these enzymes mitigate the negative impacts of anti-nutritional factors, increase digestive efficiency, and contribute to superior broiler production outcomes.

Broiler chickens rely on both endogenous and exogenous enzymes for effective feed digestion. Endogenous enzymes, produced naturally in the gastrointestinal tract, handle many dietary components adequately. However, broiler diets often contain significant amounts of NSPs, which endogenous enzymes digest poorly, resulting in reduced nutrient availability, increased digesta viscosity, and impaired growth performance. Exogenous enzyme supplementation addresses this limitation by breaking down complex carbohydrates and proteins into absorbable forms, thereby improving FCR, body weight gain, and nutrient digestibility [48, 49]. Such supplementation also alleviates the anti-nutritive effects of certain feed constituents [50].

Combined application of NSP enzymes and proteases, for example, has been shown to enhance pancreatic enzyme secretion and overall nutrient digestibility [51]. Exogenous enzymes can further modify the intestinal environment, promoting better nutrient absorption and immune function. Challenges in their practical use include maintaining enzyme stability and activity during feed processing (e.g., high-temperature pelleting can reduce efficacy) [52]. Recent advances in enzyme technology have focused on developing thermostable formulations and high-activity strains better suited to modern feed processing conditions [53].

Overall, strategic supplementation with exogenous enzymes represents an effective approach to optimize nutrient utilization, enhance growth performance, improve feed efficiency, and achieve greater economic returns in broiler production, provided enzymes are carefully selected based on feed composition, processing methods, and target outcomes.

## EFFECTS OF ENZYME SUPPLEMENTATION ON GUT HEALTH AND INTESTINAL MORPHOLOGY IN BROILERS

Numerous studies have demonstrated that enzyme supplementation plays a key role in improving the intestinal microbial balance of broilers. Zhang *et al*. [54] showed that mannanase supplementation promotes the proliferation of beneficial bacteria, such as *Lactobacillus* and *Bifidobacterium*, in the ileum while limiting the adhesion and colonization of pathogenic microorganisms, including *Escherichia coli* and *Salmonella*, on the intestinal epithelium. Similarly, Zhang *et al*. [55] reported that enzymes derived from Trichoderma reesei modulate gut microbiota diversity. At the genus level, these enzyme-supplemented diets significantly reduced the relative abundance of Bacteroides and increased the abundance of Parabacteroides—which supports mucosal immune regulation, reduces inflammation, and participates in carbon metabolism—and Faecalibacterium, which helps mitigate intestinal inflammation.

The precise mechanisms by which enzymes improve microbial balance are not fully elucidated, but they likely involve regulation of nutrient flow to the microbiota and provision of specific fermentable substrates that act as prebiotics [54, 56]. Non-starch polysaccharide-degrading enzymes (NSPase), phytases, and proteases enhance host digestion and absorption of nutrients in the upper gastrointestinal tract. This reduces the amount of undigested protein and starch reaching the lower intestine (ileum and ceca), thereby limiting substrates available to potentially harmful bacteria. By restricting protein flow while supplying fermentable carbohydrates, enzymes favor carbohydrate fermentation and the production of beneficial short-chain fatty acids (SCFA), such as butyrate, rather than protein putrefaction [56]. In terms of prebiotic effects, Zhang *et al*. [54] observed that mannanase hydrolyzes mannan into mannan-oligosaccharides (MOS), which function as prebiotics. Mannanase supplementation also alters the intestinal environment by generating SCFA, contributing to a healthier and more stable microbial community.

In addition to modulating microbiota, enzyme supplementation improves intestinal morphology and barrier integrity in broilers. Zhang *et al*. [54] found that mannanase supplementation reduced crypt depth and increased the villus height to crypt depth ratio in the jejunum and ileum. These improvements are likely linked to the anti-inflammatory properties of mannanase, which help maintain and enhance villus and crypt structure. Mannanase also strengthens intestinal barrier integrity, which is essential for preventing pathogen and toxin translocation into the bloodstream. In this context, mannanase significantly upregulated the expression of key tight junction proteins, including claudin-1 (CLDN1) and zonula occludens-1 (ZO-1), in the intestine [54]. Consistent with these findings, Bedford and Apajalahti [56] reported that enzymes enhance intestinal morphology and health by improving nutrient digestion, modulating substrate availability for gut microbiota, optimizing microbial composition, reducing inflammation, and increasing production of beneficial metabolites such as SCFA.

Enzymes are incorporated into broiler diets either as single-enzyme preparations or as multi-enzyme complexes. Some studies report similar effects between single and multi-enzyme forms, while others highlight differences. Kwak *et al*. [8] evaluated male broilers fed distillers dried grains with solubles (DDGS)-based diets supplemented with either mannanase alone or a multi-enzyme complex (mannanase, xylanase, and glucanase). Both approaches improved gut health and growth performance, including dry matter, crude protein, and gross energy digestibility [8]. However, multi-enzyme complexes often provide more comprehensive benefits. In the same study, the multi-enzyme combination significantly reduced populations of pathogenic bacteria, coliforms, and Bacteroidetes, while improving gut mucus-secreting cells and reducing inflammatory cytokines in the jejunum [8]. Overall, while single enzymes and multi-enzyme complexes can both enhance performance, multi-enzyme preparations appear to offer broader advantages, particularly for gut health and nutrient utilization. Multi-enzymes target multiple anti-nutritional factors across diverse feed ingredients, thereby optimizing growth and health more effectively [8, 57]. The choice between single- and multi-enzyme supplementation should be guided by diet composition and specific production goals [8, 58, 59].

Incorporating enzymes into poultry feed is an effective strategy to improve digestion, but it must comply with stringent regulatory requirements to ensure safety and efficacy. Regulatory authorities, such as the Food and Drug Administration (FDA) in the United States and the European Food Safety Authority (EFSA) in the European Union, oversee the approval process. Comprehensive data on manufacturing processes, toxicological testing, and efficacy are required to confirm that enzymes are safe for target animals, consumers, and the environment. Enzymes must demonstrate a clear capacity to enhance nutrient digestion or feed efficiency before approval is granted [60]. Regulatory guidelines also stress the importance of administering appropriate dosages to maximize benefits while avoiding any potential adverse effects.

## ENZYME PRODUCTION FROM AGRO-INDUSTRIAL BYPRODUCTS

The selection of substrates is a critical factor in enzyme production. Utilizing agro-industrial byproducts as low-cost substrates has emerged as an economically viable and environmentally sustainable strategy for manufacturing essential industrial enzymes [61]. This approach not only reduces raw material costs but also contributes to waste recycling and mitigation of environmental pollution by converting residues into high-value products.

Agro-industrial byproducts serve as inexpensive, renewable, and nutrient-rich feedstocks for enzyme production. A diverse array of enzymes can be synthesized from these materials, including hydrolytic enzymes such as cellulase and xylanase, which play key roles in cellulose and hemicellulose degradation [62]. Amylase production has been successfully achieved using substrates such as wheat bran and potato peel [63]. Lipolytic enzymes (e.g., lipases) can be produced by fungi such as *Aspergillus niger* through SSF [64]. Pectinolytic enzymes, widely used in fruit juice processing, are effectively generated from fruit processing wastes via SSF [65].

Enzyme production is primarily carried out through microbial fermentation involving bacteria, yeasts, or fungi. The two principal fermentation techniques employed are submerged fermentation (SmF) and SSF. These methods differ markedly in terms of yield, process efficiency, operational conditions, and suitability for different substrates. SSF is generally preferred for enzyme production from agro-industrial byproducts due to the physical-chemical compatibility of many lignocellulosic substrates with solid-phase cultures. SSF mimics the natural habitat of many filamentous fungi, requires lower water input, generates higher enzyme concentrations, and often results in reduced downstream processing costs compared to SmF [66].

In addition to conventional SmF and SSF, novel non-conventional approaches, including on-site enzyme production, have been developed and validated to further improve yield and efficiency. On-site enzyme production involves synthesizing enzymes directly at or near the point of application (e.g., within feed mills or poultry farms), thereby eliminating transportation, storage, and associated costs. This strategy integrates enzyme production into the local value chain and can utilize either SSF or SmF depending on the feedstock and infrastructure available [24].

The compositional diversity and nutrient profile of agro-industrial byproducts—such as sugar cane bagasse, corn cob, rice bran, wheat bran, and fruit peels—make them highly suitable for enzyme production. Pretreatment of substrates (e.g., chemical, thermal, or biological methods) has been shown to significantly enhance enzyme yields by several folds by improving substrate accessibility and reducing inhibitory compounds [66]. Combining different substrates or supplementing with co-substrates can further optimize production. For example, mixing rice bran with glycerol has been reported to substantially increase lipase production [64].

Overall, leveraging agro-industrial byproducts for enzyme production aligns with circular economy principles, offering cost-effective, eco-friendly alternatives to traditional substrates while supporting sustainable enzyme supply chains for applications in animal nutrition, particularly broiler feed supplementation.

## ENZYMATIC TREATMENT TO IMPROVE THE FUNCTIONAL AND NUTRITIVE VALUES OF AGRO-INDUSTRIAL BYPRODUCTS

In addition to serving as substrates for enzyme production, agro-industrial byproducts can be directly subjected to in vitro enzymatic treatment to enhance their functional and nutritive properties while reducing environmental pollution. Materials such as grape residues, wheat bran, sugarcane bagasse, citrus fruit wastes, longan peel, and feather meal are abundant, rich in organic compounds, and offer significant potential for enzyme-assisted bioprocessing to convert them into high-value feed ingredients or bioactive sources [23, 24].

Enzyme-assisted extraction has proven particularly effective for releasing bound bioactive compounds, especially antioxidative phenolics, from various residues. Commercial enzyme preparations, including Celluclast® 1.5 L (cellulase-rich), Pectinex® Ultra (pectinase-rich), and Novoferm® (a combination primarily of cellulases, pectinases, and laccases), have been successfully applied to hydrolyze grape pomace, liberating phenolic compounds such as o-coumaric acid. These treatments markedly increased total phenolic content and antioxidant capacity, as measured by Folin-Ciocalteu and 2,2-diphenyl-1-picrylhydrazyl (DPPH) assays, with Novoferm® showing particularly high efficiency [23]. Similarly, treatment of longan peel waste with cellulase and β-glucosidase significantly improved phenolic extraction yields and antioxidant activity [67]. Microbial enzymes are especially promising because they efficiently degrade cell wall components, thereby releasing bound phenolics that are otherwise inaccessible [68]. Oxidoreductases, such as laccase, tyrosinase, and horseradish peroxidase, also play important roles by converting phenolic pollutants into less toxic or more valuable derivatives, supporting sustainable production of functional compounds [69].

Enzymatic treatment is equally valuable for processing protein-rich byproducts. Feather meal, a keratin-rich waste from the poultry industry, is notoriously resistant to degradation due to its high cysteine content, extensive disulfide bonds, and dense β-sheet structure. Keratinase treatment effectively hydrolyzes these recalcitrant proteins, improving digestibility and nutritional value. Recent studies in broiler chickens have demonstrated that keratinase-treated feather meal can replace up to 33 % of soybean meal in diets without compromising performance, offering a sustainable alternative protein source [70].

Combining fermentation with enzymatic treatment provides synergistic benefits, leveraging the broad metabolic capabilities of microorganisms alongside the targeted catalytic action of enzymes to more completely break down complex substrates and release valuable nutrients. For instance, SSF of barley combined with fibrolytic enzymes substantially increased crude protein content while reducing fiber levels, resulting in improved feed utilization and growth performance in broilers [71]. More recently, Stringari *et al*. [72] showed that integrating enzymatic hydrolysis (using amylase, amyloglucosidase, and protease) with fermentation (using Lactobacillus plantarum and Saccharomyces cerevisiae) effectively valorized bread waste. Enzymatic pretreatment degraded starch and proteins into more accessible carbon and nitrogen sources for the fermenting microbes, while fermentation further enhanced the biochemical profile by increasing maltose, lactic acid, and peptide concentrations and reducing free amino acid levels. This combined approach improved both the nutritional quality and functional properties of the byproduct, demonstrating an efficient and sustainable strategy for food waste utilization in animal feed.

Overall, enzymatic treatment, whether applied alone or in combination with microbial fermentation, offers a powerful, eco-friendly method to upgrade the nutritive and functional value of agro-industrial byproducts. By improving digestibility, bioavailability of bioactive compounds, and overall feed quality, these bioprocesses support higher inclusion levels in broiler diets, reduce reliance on conventional ingredients, and advance circular bioeconomy principles in poultry production.

## IMPACTS OF ENZYME SUPPLEMENTATION ON THE OPTIMIZATION OF AGRO-INDUSTRIAL BYPRODUCT USE IN BROILER CHICKENS

Numerous studies have investigated the incorporation of agro-industrial byproducts as alternative energy and protein sources in broiler chicken diets, with enzyme supplementation playing a pivotal role in optimizing their utilization (Table 1) [8–10, 50, 73–83]. Enzyme addition consistently enables higher inclusion levels of these byproducts without compromising performance, often improving growth parameters, feed efficiency, and nutrient digestibility.

**Table 1:** Studies on enzyme supplementation to optimize the use of agro-industrial byproducts in broiler diets.

Enzymes and their levels in feeds	Agro-industrial byproducts and their levels in feeds	Enzyme action in broiler chickens	Performance gain	Economic benefits	Reference
Multienzyme complex (mannanase, xylanase, and glucanase) at 0.10 % of feed	DDGS at 5 % during starter phase and 10 % during grower and finisher phases	Enzyme increased weight gain (by 7.42 %) and feed intake (11.17 %) and decreased FCR (3.51 %) of broilers fed 10 % DDGS during finisher phase compared to 5 % without enzyme	Weight gain ↑ 7.42 %, feed intake ↑ 11.17 %, FCR ↓ 3.51 %	Not mentioned	[8]
Polyzyme® at 0.04 % of the feed	Sweet OPM at 20 % and 25 % of the feed	Enzyme increased final body weight (by 2.42 %), weight gain (2.44 %), and feed intake (3.40 %) of broilers fed 25 % sweet OPM compared with 20 % without enzyme	Body weight ↑ 2.42 %, weight gain ↑ 2.44 %, feed intake ↑ 3.40 %	Not mentioned	[9]
Multicarbohydrase complex + phytase at 0.01 % of the feed	DDGS at 0 %, 7 %, and 14 % in feeds	Enzyme optimized DDGS use up to 14 % with no adverse effects on growth and intestinal health (no differences in weight gain, feed intake, and FCR among groups)	No negative impact; maintained performance	Not mentioned	[10]
Allzyme® SSF (containing protease, amylase, β-glucanase, xylanase, pectinase, cellulase, and phytase) at 0.02 %/kg feed	Canola meal at 20 % in the diet	Enzyme increased feed intake (by 5.17 %) and body weight gain (8.59 %) and decreased FCR (3.20 %) in broilers fed 20 % canola meal compared with 20 % without enzyme	Feed intake ↑ 5.17 %, body weight gain ↑ 8.59 %, FCR ↓ 3.20 %	Not mentioned	[50]
Avizyme® 1500 (mixture of xylanase, protease, and amylase) at 0.1 g/kg	Sunflower seed meal at 25 %, 50 %, and 75 % replacing soybean meal	Enzyme increased weight gain (by 6.2 %) and α-amylase activity (16.4 %) and decreased feed intake (1.6 %) and FCR (6.4 %) of broilers fed 50 % sunflower seed meal replacing soybean meal compared with the same diet without enzyme	Weight gain ↑ 6.2 %, FCR ↓ 6.4 %	Not mentioned	[73]
Maxigrain® at 100 mg/kg of feed	Brewers’ dried grain at 0 %, 5 %, 10 %, and 15 % of diets	Enzyme increased daily weight gain (by 15.8 %) and feed intake (9.0 %), while decreasing FCR (6.12 %) of broilers fed 15 % brewers’ dried grain compared to 15 % without enzyme	Daily weight gain ↑ 15.8 %, feed intake ↑ 9.0 %, FCR ↓ 6.12 %	Reduced cost per kg weight gain by 6.77 % at 15 % brewers’ dried grain	[74]
Natuzyme P50® at 0, 350, and 700 ppm	Dried orange peel powder at 0 %, 2 %, and 4 % of feeds	Enzyme increased body weight gain (by 8.36 %) of broilers fed 4 % dried orange peel powder	Body weight gain ↑ 8.36 %	Not mentioned	[75]
Multiblend enzymes (xylanase, β-glucanase, cellulase, α-amylase, protease, and phytase) at 0.05 % of the diet	Palm kernel cake at 10 % and 20 % of the diet	Enzyme increased fasted live weight (by 6.47 %) of Sasso broilers fed 20 % palm kernel cake	Fasted live weight ↑ 6.47 %	Not mentioned	[76]
Xylanase, amylase, and protease multi-enzymes at 50 g/kg of feed	Yam peel meal at 15 % and 30 % of the diet	Enzyme increased weight gain (by 1.05 %) and feed intake (2.17 %) of broilers fed 30 % yam peel meal compared with 30 % without enzyme	Weight gain ↑ 1.05 %, feed intake ↑ 2.17 %	Highest profit margin at 30 % yam peel meal with enzyme	[77]
Enzyme mixture (xylanase, cellulase, β-glucanase, pectinase, amylase, protease, and phytase) at 250 g/ton of feed	Potato peels at 15 % of the feeds	Enzyme increased digestibility of dry matter (by 8.58 %), organic matter (8.46 %), crude protein (9.93 %), crude fat (9.42 %), and crude fiber (55.7 %) with no adverse effect on small intestinal development and meat quality	Dry matter digestibility ↑ 8.58 %, crude protein ↑ 9.93 %, crude fiber ↑ 55.7 %	Not mentioned	[78]
Enzyme mixture (xylanase, cellulase, β-glucanase, pectinase, amylase, protease, and phytase) at 250 g/ton of feed	Sugar beet pulp at 7.5 % of feed	Enzymes increased digestibility of dry matter (9.63 %), organic matter (11.7 %), crude protein (13.9 %), crude fat (12.7 %), crude fiber (103 %), and ash (3.35 %) with no adverse effect on small intestinal development and meat quality	Dry matter digestibility ↑ 9.63 %, crude protein ↑ 13.9 %, crude fiber ↑ 103 %	Not mentioned	[78]
Nutrizyme® at 0.25 % of the diet	Cassava peel meal and palm kernel cake mixture at 25 % and 50 % of the diets	Enzyme increased body weight gain (by 9.27 %) and decreased feed intake (18.8 %) and FCR (25.8 %) in broilers fed 50 % cassava peel meal and palm kernel cake mixture	Body weight gain ↑ 9.27 %, FCR ↓ 25.8 %	Not mentioned	[79]
Multi-enzymes (phytase, cellulase, and xylanase) at 0 and 0.5 g/kg	Olive pomace at 0 %, 5 %, and 10 % of diets	Enzyme increased feed consumption (by 7.76 %) and body weight gain (8.57 %) and decreased FCR (0.83 %), while improving serum lipid profile and liver enzyme activity of broilers fed 10 % olive pomace	Feed consumption ↑ 7.76 %, body weight gain ↑ 8.57 %, FCR ↓ 0.83 %	Not mentioned	[80]
Protease at 200 mg/kg or phytase at 100 mg/kg	Corn-DDGS at 30 % of the diet	Enzymes increased metabolizability of dry matter (protease by 5.41 %, phytase by 7.68 %) and crude protein (both by 28.7 %) and amino acid digestibility (protease by 7.3 %)	Dry matter metabolizability ↑ 5.41–7.68 %, crude protein ↑ 28.7 %	Not mentioned	[81]
Xylanase at 100 ppm	Palm kernel cake at 10 %, 20 %, or 30 % replacing maize	Enzyme increased weight gain (by 8.97 %) and decreased feed intake (3.14 %) and FCR (11.5 %) in broilers fed 30 % palm kernel cake	Weight gain ↑ 8.97 %, FCR ↓ 11.5 %	Reduced feed costs by 12.05 % at 30 % palm kernel cake	[82]
NSPase enzyme combination at 0.5 kg/ton of diet	Sunflower meal at 15 %, 20 %, and 25 % of the diet	Enzymes increased weight gain (by 0.96 %), decreased feed intake (5.02 %), and FCR (6.32 %) of broilers fed 20 % sunflower meal	Weight gain ↑ 0.96 %, FCR ↓ 6.32 %	Not mentioned	[83]

Corn-DDGS = Corn distillers dried grains with soluble, DDGS = Distillers dried grains with soluble, FCR = Feed conversion ratio, NSPase = Non-starch polysaccharide-degrading enzyme, OPM = Orange peel meal. All percentage changes are relative to the corresponding unsupplemented control group within each study.

For DDGS, exogenous enzyme supplementation significantly enhances weight gain, FCR, and nutrient digestibility compared to unsupplemented diets [8]. This allows DDGS to be included at greater proportions without adverse effects. Dal Pont *et al*. [10] demonstrated that a multicarbohydrase complex (containing xylanase, β-glucanase, arabinofuranosidase, and phytase) optimized DDGS inclusion up to 14 % (versus 7 %), resulting in improved FCR and body weight gain in broilers at 21 days of age.

Enzyme supplementation also supports higher replacement levels of soybean meal with sunflower seed meal. Dietary addition of Avizyme® 1500 (a mixture of xylanase, protease, and amylase) at 0.1 g/kg enabled sunflower seed meal to replace soybean meal up to 50 % (compared to 25 % without enzyme) [73]. Broilers fed 50 % sunflower seed meal with enzyme exhibited improved body weight, weight gain, and FCR (1.61 versus 1.72 and 1.68 for unsupplemented 50 % and 25 % levels, respectively). Enzyme supplementation further increased amylase and protease activities, facilitating better nutrient digestion and utilization.

In the case of brewers’ dried grain, enzyme supplementation (e.g., Maxigrain® at 100 mg/kg) optimized inclusion up to 15 % (compared to 5 % and 10 % without enzyme) while enhancing performance [74]. The 15 % inclusion level with enzyme not only maintained or improved growth but also reduced production cost per kilogram of weight gain, highlighting economic advantages.

Sweet OPM inclusion can be increased to 25 % with Polyzyme® supplementation without negatively affecting final body weight, weight gain, feed intake, or protein digestibility, whereas unsupplemented 25 % levels reduced these parameters compared to 20 % [9]. Enzyme addition reversed the decline in crude protein utilization observed at higher inclusion rates. Similarly, Natuzyme P50® improved body weight gain in broilers fed 4 % dried orange peel powder, with enzyme-supplemented groups (at 350 ppm and 700 ppm) outperforming unsupplemented 4 % and even 2 % levels [75].

Palm kernel cake utilization also benefits from enzyme treatment. Supplementation with enzyme mixtures increased inclusion from 10 % to 20 % without major negative impacts [84], while mannanase-treated palm kernel meal allowed substitution of corn up to 25 % (versus 12.5 % for untreated) without compromising performance or carcass quality [85]. The enzyme degrades mannans, improving digestibility.

Enzyme supplementation generally increases byproduct inclusion by enhancing digestibility of NSPs and mitigating anti-nutritional factors. β-Glucanase and xylanase break down viscous fibers in high-fiber byproducts such as olive cake and palm kernel cake, improving nutrient absorption [86]. Phytase reduces phytic acid’s anti-nutritional effects, enhancing phosphorus availability and supporting higher inclusion rates [86]. Enzyme mixtures have also been shown to elevate serum albumin levels, reflecting improved protein status and correlating with better growth performance [84].

In summary, enzyme supplementation significantly expands the safe and effective inclusion levels of agro-industrial byproducts in broiler diets, improving nutrient utilization, performance, and economic viability. Success relies on matching enzyme type(s) to the specific anti-nutritional profile of the byproduct, with multi-enzyme complexes often providing superior results in heterogeneous diets. Careful selection is essential to maximize benefits while avoiding suboptimal outcomes.

Enzyme supplementation can significantly increase the inclusion level of agro-industrial byproducts in broiler diets by improving digestibility and nutrient utilization. This enables higher proportions of these byproducts without compromising broiler health and performance [76]. Enzymes such as β-glucanase and xylanase enhance the digestibility of NSPs, thereby increasing absorption and utilization of nutrients from high-fiber byproducts such as olive cake and palm kernel cake through the breakdown of complex polysaccharides and improved nutrient accessibility [86]. Phytase similarly mitigates antinutritional factors by enhancing phosphorus availability and reducing the adverse effects of phytic acid, supporting higher inclusion rates of byproducts like olive cake while maintaining health and growth [86]. Zubaidah *et al*. [84] further reported that supplementation with an enzyme mixture (NSPase at 400 g/ton, protease at 260 g/ton, and mannanase at 182 g/ton) increased serum albumin levels, a key indicator of protein status and overall health that positively correlates with improved growth performance in broilers.

In contrast, Stokvis *et al*. [87] found that proteolytic enzyme treatment did not improve production performance and actually reduced nutrient digestibility in broilers fed co-products of Ulva laetevirens and Solieria chordalis. Uns upplemented inclusion of these seaweed co-products increased FCR, decreased apparent pre-cecal digestibility of dry matter, organic matter, nitrogen, and all amino acids, and reduced duodenal villus height. The authors recommended future research on carbohydrase enzymes to target the unique polysaccharides in seaweed co-products, which may improve nutrient availability and counteract the observed negative effects. These findings highlight that enzyme type and specificity must be carefully matched to the compositional characteristics of the agro-industrial byproduct to achieve optimal results.

While both single-enzyme and multi-enzyme preparations are used in diets containing agro-industrial byproducts, the choice depends on dietary composition and production objectives [8]. Single enzymes target specific substrates—for example, breaking down a particular fiber type or protein fraction—and can be effective when the diet is dominated by one major indigestible component [88]. Multi-enzyme complexes, however, address a wider range of anti-nutritional factors simultaneously, making them more suitable for complex diets with diverse indigestible components [6, 88]. The combined action of multiple enzymes improves the breakdown of various fibers and proteins, thereby enhancing overall nutrient bioavailability [6]. Consequently, multi-enzyme preparations often provide superior benefits by comprehensively improving the nutritional profile of byproduct-based diets [89].

Enzymes may also be combined with other feed additives to further enhance their effectiveness. Alderey *et al*. [90] demonstrated that combining econase with a probiotic (dry yeast) maximized the benefits of sieved olive pulp in laying hen diets. However, synergistic effects are not universal in broilers. Yaseen *et al*. [91] found that combining protease with organic acids provided no additional benefit and negatively affected outcomes in diets containing poultry byproduct meal. Protease alone significantly improved FCR across the experimental period compared to the control, whereas the combined supplementation resulted in FCR similar to the unsupplemented group. Crude protein digestibility was highest in the protease-only and control groups but lowest in the combined group. These results indicate that combining enzymes with other additives requires careful evaluation, as inappropriate combinations may diminish rather than enhance the ability to maximize agro-industrial byproduct use as alternative feed ingredients.

In conclusion, enzyme supplementation substantially improves the practical and nutritional value of agro-industrial byproducts in broiler production by enabling higher inclusion levels, enhancing nutrient utilization, and supporting better performance and economic outcomes. The effectiveness depends on precise matching of enzyme(s) to byproduct composition, with multi-enzyme complexes frequently offering broader advantages in diverse diets. Strategic selection and judicious combination with other additives are essential to fully realize these benefits while avoiding potential counterproductive interactions.

## EFFECTS OF ENZYME SUPPLEMENTATION ON THE ECONOMIC PERFORMANCE OF BROILERS FED AGRO-INDUSTRIAL BYPRODUCTS

From an economic perspective, incorporating agro-industrial byproducts as alternative feed ingredients can substantially reduce feed costs in broiler production (Table 1) [74, 77, 82]. This cost-saving potential is especially important for maintaining industry sustainability amid rising prices of conventional ingredients such as corn and soybean meal. Enzyme supplementation further enhances these economic benefits by improving feed efficiency and performance, often offsetting the added cost of the enzymes themselves.

Okukpe *et al*. [92] showed that including palm kernel cake in broiler diets lowered overall feed costs. However, increasing palm kernel cake levels without enzyme supplementation raised the cost per kilogram of broiler produced, reducing profitability. Adding xylanase slightly increased initial feed cost but decreased the total cost of raising 1 kg of broiler due to improved feed efficiency and weight gain. Aderibigbe *et al*. [82] conducted a cost-benefit analysis and reported that 30 % palm kernel cake inclusion reduced feed costs by up to 15.53 % without enzyme. With xylanase supplementation, feed cost reduction was 12.05 % at the same inclusion level, yet the total rearing cost per kilogram of broiler decreased across all inclusion levels. The most favorable outcome occurred at 10 % palm kernel cake with xylanase, combining significant cost reduction with markedly improved performance.

Oguntoye *et al*. [77] found that enzyme supplementation in yam peel meal diets increased both the cost per kilogram of diet and the cost per kilogram of weight gain. Nevertheless, the enzyme-supplemented diet (particularly at 30 % yam peel meal) achieved the highest gross profit margin. This was attributed to moderate diet costs combined with superior growth performance, demonstrating that improved FCR and weight gain can outweigh modest increases in feed cost and deliver the greatest net economic return.

Aku *et al*. [74] similarly reported economic advantages with brewers’ dried grain. Feed cost per kilogram decreased as inclusion level increased. Supplementation with Maxigrain® (100 mg/kg) further reduced production costs. Although total feed consumed per bird increased slightly with enzyme, the cost per kilogram of weight gain was lowest at 15 % brewers’ dried grain with enzyme. The improved FCR in enzyme-supplemented diets was a key driver of this economic benefit, reflecting better nutrient utilization and lower overall production expense.

While these findings are highly promising, the economic advantages of enzyme supplementation in byproduct-based diets must be interpreted cautiously. Most studies have been conducted under controlled laboratory conditions with limited bird numbers, leading to potential variability in results. Differences in byproduct quality, enzyme formulations, inclusion levels, bird strains, management practices, and local feed prices may influence outcomes when scaled to commercial operations. The gap between experimental settings and real-world farm conditions underscores the need for large-scale, on-farm trials to validate these economic benefits and enable broiler producers to confidently adopt enzyme-assisted byproduct utilization strategies.

In summary, enzyme supplementation often transforms agro-industrial byproducts from cost-neutral or costly alternatives into economically advantageous feed components by enhancing performance metrics such as FCR that directly reduce the cost per kilogram of broiler produced. Strategic use of enzymes can therefore support greater sustainability and profitability in broiler production, provided future research bridges the laboratory-to-commercial divide.

## EFFECTS OF ENZYME SUPPLEMENTATION ON THE INTESTINAL HEALTH OF BROILERS FED AGRO-INDUSTRIAL BYPRODUCTS

While agro-industrial byproducts can positively influence intestinal microbial balance [10, 93] and stress resilience [93] in broiler chickens, their inclusion often negatively affects intestinal immune function and morphology. Dal Pont *et al*. [10] reported that short-term feeding (up to 14 days) with 7 % or 14 % DDGS had no significant impact on intestinal health, but prolonged exposure led to adverse changes in the intestinal immune system. These negative effects were linked to morphological alterations, including thicker intestinal layers/lamina propria and increased infiltration of inflammatory cells into the lamina propria and epithelium [10]. Zubaidah *et al*. [84] similarly observed that increasing palm kernel cake from 10 % to 20 % raised crypt depth in the duodenum and ileum while reducing villus height in the jejunum, potentially impairing small intestinal absorptive capacity. Abdel-Daim *et al*. [78] noted that sugar beet pulp, particularly during the starter phase, reduced villus height and villus height to crypt depth ratio, likely due to increased digesta viscosity in young birds that hindered nutrient utilization. High levels of tannins in grape pomace also caused histological lesions in the ileum, reduced crypt depth, and decreased intestinal wall thickness, leading to lower villus height and villus height to crypt depth ratio compared to controls [93].

Enzyme supplementation effectively counteracts many of these adverse effects on intestinal immune function and morphology. Dal Pont *et al*. [10] demonstrated that a multicarbohydrase complex combined with phytase mitigated the negative immune impacts of DDGS by reducing immune cell infiltration in the duodenal, jejunal, and ileal epithelium and decreasing inflammatory cell presence in the ileal lamina propria. The enzyme combination also modulated the intestinal microbiota, lowering dysbiosis-associated Proteobacteria and *Enterococcaceae*, thereby enabling safe use of higher DDGS levels [10]. Okukpe *et al*. [92] found that xylanase supplementation in palm kernel cake diets promoted beneficial microbes (*Lactobacillus* and *Bifidobacterium*) while suppressing pathogenic bacteria and fungi. This improvement resulted from the enzyme’s hydrolysis of NSPs, releasing prebiotic oligosaccharides that serve as substrates for beneficial microorganisms. Zubaidah *et al*. [84] showed that an enzyme mixture (mannanase, NSPase, and protease) alleviated palm kernel cake-induced morphological changes by increasing villus height and width in the duodenum and ileum and reducing crypt depth in the duodenum. Abdel-Daim *et al*. [78] confirmed that enzyme mixtures (e.g., Enziver®) mitigated sugar beet pulp’s negative effects on intestinal morphology by reducing viscosity, degrading viscous fibers, and improving nutrient accessibility, which supported better development of absorptive structures and overall gut health.

Beyond morphology and microbiota, enzyme supplementation enhances stress resilience in broilers fed agro-industrial byproducts. Ebrahimzadeh *et al*. [93] reported that adding tannase (1000 mg/kg) to 10 % grape pomace diets increased activities of superoxide dismutase (SOD) and glutathione peroxidase (GSH-Px) while decreasing serum malondialdehyde (MDA) levels, the lowest MDA and highest antioxidant enzyme activities were observed in this group compared to others, indicating reduced oxidative stress.

Enzyme supplementation also contributes to broader health and sustainability goals. Antimicrobial resistance in broilers, driven by overuse of antimicrobial growth promoters to support gut health, poses risks to human health via the food chain [94]. By improving intestinal health and function, enzymes offer an effective alternative that can reduce or eliminate the need for antibiotic growth promoters and therapeutic antibiotics, particularly in byproduct-based diets. Moreover, maximizing agro-industrial byproduct use decreases reliance on corn and soybean meal, major contributors to greenhouse gas (GHG) emissions and climate change [95], thereby lowering the broiler industry’s overall carbon footprint and supporting climate change mitigation efforts.

In conclusion, while certain agro-industrial byproducts can impair intestinal immune function, morphology, and oxidative status when fed at higher levels, strategic enzyme supplementation largely reverses these negative effects, restores gut health parameters, enhances stress resilience, reduces antimicrobial dependency, and promotes environmental sustainability. These benefits reinforce the value of enzyme-assisted strategies for safe, efficient, and eco-friendly incorporation of agro-industrial byproducts in broiler nutrition.

## EFFECTS OF ENZYME SUPPLEMENTATION ON CARCASS TRAITS AND MEAT QUALITY OF BROILERS FED AGRO-INDUSTRIAL BYPRODUCTS

Feeding agro-industrial byproducts without enzyme supplementation frequently impairs carcass traits and meat quality in broiler chickens. Alefzadeh *et al*. [75] reported that inclusion of 2 % dried orange peel powder reduced weights of most valuable carcass parts, with more pronounced effects at 4 %. Supplementation with the multi-enzyme Natuzyme P50® (700 ppm) mitigated these negative impacts, particularly supporting development of prime cuts such as drumsticks. Ukorebi [79] observed that diets containing dried cassava peel and palm kernel cake adversely affected dressed weight, although some prime cuts (thigh/drumstick) remained unaffected. Nutrizyme® supplementation improved digestion of high-fiber components, leading to higher final body weights, increased dressed weight, higher dressing percentage, and greater weights of prime cuts [79].

Okukpe *et al*. [92] found that increasing palm kernel cake levels significantly reduced weights of commercially valuable parts, including thighs, drumsticks, and breast. Koranteng *et al*. [76] showed that raising palm kernel cake from 10 % to 20 % decreased dressing percentage in Sasso broilers. Multiblend enzyme supplementation increased leg and drumstick weights, likely due to improved FCR and heavier body weight. Xylanase supplementation also reduced abdominal fat deposition across palm kernel cake levels by enhancing nutrient utilization and limiting fat storage [92]. The enzyme mixture further improved carcass yield by degrading antinutritional factors and indigestible fibers in palm kernel cake, unlocking nutrients and promoting muscle synthesis, particularly in the breast [76].

Negative effects extend to meat quality. Palm kernel cake inclusion resulted in tougher texture (higher shear force) and lower redness in breast meat [96]. Xylanase supplementation lowered shear force values, indicating more tender meat, and increased redness of breast meat. These improvements were attributed to better nutrient digestibility through breakdown of NSPs and antinutritional factors, which primarily enhance growth but indirectly support favorable meat characteristics [96].

In contrast, enzyme supplementation was ineffective in some cases. Inclusion of 8 % guar meal (a byproduct of guar gum production) reduced dressing percentage by 2 %, decreased carcass muscularity by 3 %, increased saturated fatty acids (myristic C14:0 and palmitic C16:0), and worsened the n-6:n-3 PUFA ratio, atherogenic index (AI), and thrombogenic index in muscles compared to controls [97]. Enzyme preparations containing β-glucanase, hemicellulase, and pentosanase, or α-galactosidase and β-glucanase, had no effect on carcass traits or meat quality. The lack of benefit was likely due to other antinutritional factors in guar meal (e.g., trypsin inhibitors, saponins, polyphenols) beyond β-mannan, underscoring the need to select enzymes matched to the specific profile of the byproduct.

Agro-industrial byproducts may also introduce mycotoxins (aflatoxins, ochratoxins, zearalenone, fumonisins) from fungal contamination (*Aspergillus*, *Fusarium*, *Penicillium*), which impair nutrient absorption, reduce growth, and compromise meat safety and quality [98, 99]. Certain enzymes mitigate mycotoxin risks by transforming them into less toxic or non-toxic metabolites via acetylation, glucosylation, hydrolysis, or deamination [100, 101]. Such enzymatic detoxification reduces mycotoxin bioavailability in the gastrointestinal tract, limiting uptake and accumulation in tissues, thereby improving broiler health, performance, and meat safety for human consumption [100]. To date, however, no studies have specifically examined the effects of enzyme supplementation on meat safety parameters in broilers fed mycotoxin-contaminated agro-industrial byproducts.

In conclusion, enzyme supplementation largely counteracts the adverse effects of agro-industrial byproducts on carcass traits (e.g., dressing percentage, prime cut weights, abdominal fat) and meat quality (e.g., tenderness, color, fatty acid profile) by enhancing nutrient utilization and degrading antinutritional factors. Effectiveness depends on enzyme specificity to the byproduct’s composition. Enzyme-assisted strategies also offer potential for mycotoxin mitigation, supporting safer meat production. These benefits reinforce the role of targeted enzyme use in enabling higher, more sustainable inclusion of agro-industrial byproducts while preserving or improving carcass and meat quality parameters essential for market value and consumer acceptance.

## EFFECTS OF ENZYME-TREATED AGRO-INDUSTRIAL BYPRODUCTS IN BROILER FEED ON CARBON FOOTPRINT REDUCTION, WATER USE, AND CIRCULAR ECONOMY

The broiler chicken industry contributes significantly to global carbon emissions, with feed production (including crop cultivation and processing) accounting for the largest share of the carbon footprint, followed by on-farm energy use for heating and ventilation, and manure management [102]. Integrating enzyme supplementation into diets based on agro-industrial byproducts offers substantial potential to reduce the carbon footprint of broiler production through improved resource efficiency and environmental sustainability. Agro-industrial byproducts are abundant, renewable, and often underutilized, making their repurposing as broiler feed an effective strategy for waste reduction and mitigation of environmental impacts [103].

Enzyme supplementation enhances nutrient digestibility and bioavailability in byproduct-based diets by breaking down complex carbohydrates, proteins, and fibers (e.g., NSPs, phytate, and mannans), thereby increasing nutrient availability to broilers. This leads to improved growth performance, higher feed efficiency, and reduced total feed intake per unit of body weight gain. Since feed production is the dominant contributor to the carbon footprint in poultry systems, decreasing reliance on conventional high-emission ingredients such as corn and soybean meal—whose cultivation involves significant land use, fertilizer application, and machinery-related emissions—directly lowers the overall carbon intensity of broiler production [104]. A reduced FCR further amplifies these benefits, as less feed is required per kilogram of meat produced, resulting in lower cumulative emissions from feed manufacturing, transport, and associated activities [105]. Enzyme supplementation also contributes to reduced manure output and altered manure composition, decreasing emissions of methane and nitrous oxide from manure storage and application [102–104].

Beyond carbon footprint reduction, enzyme-treated agro-industrial byproducts can decrease water consumption linked to feed production. Conventional feed ingredients such as soybean and corn require substantial irrigation and rainfall during cultivation. By substituting these with enzyme-enhanced byproducts (e.g., palm kernel cake, DDGS, fruit peels, and brewers’ dried grain), which are often generated with minimal additional water input, the broiler industry reduces its indirect water footprint. This aligns with sustainable agriculture objectives that prioritize efficient resource use and minimization of water-intensive crop production [103, 105].

Enzyme supplementation also promotes broiler health through improved gut integrity, microbiota modulation, and the release of bioactive compounds (e.g., antioxidants and prebiotic oligosaccharides) from byproducts. Enhanced immunity and gut health further support better FCR and growth rates, compounding the environmental benefits by reducing overall resource demand per unit of output [105].

The approach strongly supports circular economy principles in livestock production, which emphasize waste minimization, resource reuse, and closed-loop systems. Enzyme supplementation enables valorization of agro-industrial residues—previously considered waste—into high-value feed ingredients, thereby reducing disposal problems, lowering feed costs, and decreasing dependence on virgin raw materials [106]. This practice embodies the reduce-reuse-recycle framework: agricultural and food processing byproducts are diverted from landfills or low-value uses and transformed into inputs for broiler production, creating a closed-loop system that enhances economic viability while mitigating environmental degradation [107, 108]. By integrating enzyme technology, the broiler sector advances toward more sustainable, resource-efficient, and circular models of production.

In summary, enzyme supplementation in diets containing agro-industrial byproducts reduces the carbon footprint and water footprint of broiler production through improved feed efficiency, decreased reliance on high-impact conventional ingredients, and lower emissions from feed and manure. It simultaneously advances circular economy goals by turning industrial residues into valuable resources, promoting waste minimization, resource recycling, and long-term sustainability in the poultry industry.

## ENZYME SUPPLEMENTATION TO INCREASE AGRO-INDUSTRIAL BYPRODUCT INCLUSION IN BROILER DIETS: CHALLENGES AND FUTURE PERSPECTIVES

Despite the well-documented benefits of enzyme supplementation in enhancing performance and enabling higher inclusion of agro-industrial byproducts in broiler diets, several studies have reported inconsistent or absent positive effects. Carvalho *et al*. [109] found no significant improvement from protease supplementation when animal byproduct meal served as the protein source. Abd El Latif *et al*. [58] observed that multi-enzyme addition did not permit increased inclusion of OPM without performance penalties. Al-Harthi *et al*. [110] similarly reported that an enzyme cocktail failed to improve olive cake utilization beyond control levels. Aghili *et al*. [111] demonstrated that even a complex multi-enzyme preparation (containing pectinase, proteases, endo-1,4-β-mannanase, β-mannosidase, and β-D-glucanase) could not prevent detrimental effects on growth performance when dried apple pomace was included at 12%, 16%, or 20%.

Several challenges limit the consistent success and widespread application of enzymes in byproduct-based broiler diets. The most prominent is variability in byproduct composition. Agro-industrial byproducts such as olive cake, citrus pulp, grape pomace, and apple pomace exhibit substantial differences in nutritional content, fiber fractions, and antinutritional factors depending on source, processing method, and harvest conditions [105]. This compositional heterogeneity makes it difficult to achieve reliable responses with standardized enzyme formulations. Different byproducts contain unique fiber types and antinutritional compounds that require specific enzyme activities (e.g., cellulases, hemicellulases, or pectinases) not always present in commercial multi-enzyme blends designed for general poultry diets [112, 113]. Consequently, enzyme efficacy is highly dependent on precise matching between the byproduct’s substrate profile and the enzyme’s catalytic specificity, limiting the feasibility of universal or “one-size-fits-all” enzyme solutions.

Seasonal and regional fluctuations in byproduct quality further complicate enzyme application. Variations in climate, soil conditions, and harvest timing alter chemical composition, including crude fiber, phytate, and NSP levels [114]. Elevated phytate content in certain seasons or regions reduces phytase effectiveness, while higher fiber fractions increase the demand for NSP-degrading enzymes such as β-glucanase and xylanase to maintain acceptable FCR [115, 116]. Seasonal shifts in byproduct composition also influence gut microbiota responses to enzyme treatment, as the availability of fermentable substrates for beneficial bacteria changes over time [117]. These temporal and geographic inconsistencies necessitate adaptive enzyme strategies rather than fixed formulations.

Future research should leverage omics technologies, proteomics, metabolomics, metagenomics, and transcriptomics to overcome these limitations and enable more precise enzyme-byproduct matching. Multi-omics approaches provide comprehensive insights into enzyme-substrate interactions, degradation pathways, microbial community dynamics, and metabolic outcomes during digestion of enzyme-treated byproducts [118, 119]. Proteomics can quantify enzyme expression and activity in the gastrointestinal tract, while metabolomics reveals substrate conversion efficiency and optimal conditions for maximal nutrient release [120, 121]. Metagenomics and transcriptomics elucidate how enzymes reshape gut microbiota and host gene expression in response to specific byproducts. Although omics tools have advanced understanding in other fields, their application in poultry nutrition, particularly for enzyme-optimized use of agro-industrial byproducts, remains limited and represents a critical area for future investigation.

Economic considerations also constrain adoption. While enzymes improve growth performance, nutrient digestibility, and health (references [80–83]), the added cost of enzyme formulations must be justified within the narrow profit margins of broiler production [8, 58]. Cost-benefit analyses should account for variable byproduct prices, enzyme pricing, inclusion levels, performance gains, and downstream impacts on feed and production costs. In many cases, the nutritional improvements from enzymes outweigh their expense when higher byproduct inclusion reduces reliance on costly conventional ingredients, but this balance varies by region, byproduct availability, and market conditions.

In summary, enzyme supplementation holds strong potential to increase safe and efficient inclusion of agro-industrial byproducts in broiler diets, but its effectiveness is constrained by compositional variability, seasonal quality fluctuations, enzyme specificity requirements, and economic factors. Overcoming these challenges will require tailored enzyme formulations, region- and byproduct-specific recommendations, and integration of advanced omics technologies to map substrate-enzyme interactions and microbiota responses. Large-scale commercial trials, cost-effectiveness modeling, and continued refinement of enzyme blends are essential to translate laboratory successes into reliable, economically viable strategies for sustainable broiler production.

## CONCLUSION

This review highlights the transformative potential of enzyme supplementation in valorizing agro-industrial byproducts for broiler chicken diets. Key studies demonstrate that enzymes such as phytase, protease, xylanase, β-glucanase, β-mannanase, and multi-enzyme complexes enable higher inclusion levels of byproducts like DDGS (up to 14 %), sunflower seed meal (up to 50 %), palm kernel cake (up to 25 %), sweet OPM (up to 25 %), and brewers’ dried grain (up to 15 %), without compromising growth performance. Reported improvements include 1–16 % increases in body weight gain, 2–11 % in feed intake, and 1–26 % reductions in FCR, alongside enhanced nutrient digestibility (e.g., dry matter by 5–103 %, crude protein by 9–28 %). Enzymes mitigate antinutritional factors, improve intestinal morphology (increased villus height to crypt depth ratio), promote beneficial microbiota (Lactobacillus, Bifidobacterium), reduce oxidative stress, and enhance carcass traits (e.g., higher dressing percentage, reduced abdominal fat). Economically, enzyme use lowers production costs by 7–12 % per kg of broiler, while environmentally, it reduces carbon footprints by minimizing reliance on high-emission crops and lowering manure GHG emissions. Production methods like SSF using byproducts as substrates further support sustainability.

Enzyme-assisted byproduct utilization offers broiler producers a cost-effective, sustainable alternative to conventional feeds amid fluctuating prices and supply chain vulnerabilities. By enabling higher inclusion rates, this approach reduces feed costs, alleviates food-feed competition, and promotes circular bioeconomy principles through waste valorization. Improved gut health and performance decrease antibiotic dependency, addressing antimicrobial resistance concerns, while enhanced meat quality (e.g., tenderness, fatty acid profiles) meets consumer demands for healthier products. Environmentally, it lowers water and carbon footprints, supporting climate-resilient farming. For industry adoption, tailored enzyme formulations matched to local byproduct availability can optimize outcomes, particularly in developing regions like Southeast Asia with abundant residues (e.g., palm kernel cake in Indonesia).

The review’s strengths lie in its comprehensive synthesis of diverse evidence, linking enzyme mechanisms to multifaceted outcomes including performance, health, economics, and sustainability. It draws from in vivo trials, mechanistic studies, and applied research, providing a holistic view absent in prior literature focused on isolated aspects. The emphasis on enzyme-byproduct specificity, multi-enzyme benefits, and real-world challenges enhances applicability, while highlighting omics potential bridges fundamental and practical science.

Limitations include reliance on controlled laboratory trials with small sample sizes, potentially limiting generalizability to commercial scales where variability in byproduct quality, bird strains, and management practices may alter results. Inconsistent enzyme responses across byproducts (e.g., ineffective for seaweed or guar meal) underscore the need for specificity, while economic analyses often overlook regional price fluctuations or long-term costs. Few studies address mycotoxin mitigation or meat safety, and the review’s narrative nature precludes quantitative meta-analysis due to methodological heterogeneity.

Future research should prioritize large-scale, on-farm trials to validate laboratory findings under commercial conditions, incorporating multi-omics (proteomics, metabolomics, metagenomics) to tailor enzyme formulations to specific byproducts and microbiota dynamics. Investigating novel enzyme sources (e.g., from microbial fermentation of byproducts) and combinations with additives (e.g., probiotics, organic acids) could enhance efficacy. Long-term studies on mycotoxin detoxification, antimicrobial resistance reduction, and full life-cycle assessments of carbon/water footprints are needed. Exploring genetic selection for broilers better adapted to enzyme-enhanced byproduct diets, alongside economic modeling across regions, will facilitate broader adoption.

Enzyme supplementation represents a pivotal strategy for sustainable broiler production, transforming agro-industrial byproducts from environmental liabilities into valuable feed resources that enhance efficiency, health, and profitability while mitigating climate impacts. By addressing current limitations through innovative research, this approach can drive a more resilient, circular, and eco-friendly poultry industry, ensuring food security in an era of resource constraints.

## AUTHOR’S CONTRIBUTIONS

SS: Prepared and revised the manuscript. FRH, DNA, MMS, and SSTU: Read and revised the manuscript. All authors have read and approved the final manuscript.
